# Deciphering HPAI Influenza A Virus (H5N1): Molecular Basis of Pathogenicity, Zoonotic Potential, and Advances in Vaccination Strategies

**DOI:** 10.3390/v18040410

**Published:** 2026-03-26

**Authors:** Imran Mohammad, Mohammed Ibrahim Hajelbashir, Mahmoud H. El-Bidawy, Abdulwahab Abuderman, Murtaja Satea, Abdullah M. R. Arafah, Md. Rizwan Ansari, Mahjabeen Rahmani, Mohiuddin Khan Warsi, Nawal Helmi, Mohammad Azhar Kamal

**Affiliations:** 1 Department of Basic Medical Sciences, College of Medicine, Prince Sattam bin Abdulaziz University, Al-Kharj 11942, Saudi Arabia; imran.mohammad9@gmail.com (I.M.);; 2 Department of Pediatric, College of Medicine, Prince Sattam bin Abdulaziz University, Al-Kharj 11942, Saudi Arabia; 3 College of Medicine, University of Warith Al-Anbiyaa, Karbala 56001, Iraq; 4 Department of Biotechnology, College of Commerce, Arts & Science, Patliputra University, KankarBagh, Patna 800020, India; 5 Department of Biological Sciences, College of Science, University of Jeddah, Jeddah 23890, Saudi Arabia; 6 Department of Biochemistry, College of Science, University of Jeddah, Jeddah 21589, Saudi Arabia; 7 Department of Pharmaceutics, College of Pharmacy, Prince Sattam bin Abdulaziz University, Al-Kharj 11942, Saudi Arabia

**Keywords:** HPAI virus, H5N1, zoonosis, panzootic, molecular determinants, vaccination, bird flu, One Health, clade 2.3.4.4b, biosecurity

## Abstract

The ongoing panzootic of the highly pathogenic avian influenza (HPAI) H5N1 virus, dominated by clade 2.3.4.4b, constitutes a significant global threat to wildlife, animal health, and public health. Once characterized by sporadic outbreaks, H5N1 has evolved into a sustained, year-round infection with an expanded host range that now includes numerous mammalian species. Its high pathogenicity is primarily driven by the acquisition of a polybasic haemagglutinin cleavage site, enabling systemic viral spread, alongside emerging endothelial and neurotropic properties that contribute to severe disease and high mortality in mammals. Although zoonotic transmission remains limited, H5N1 continues to accumulate mutations associated with mammalian adaptation, particularly within the haemagglutinin and polymerase complex. Notably, recent outbreaks in U.S. dairy cattle highlight the emergence of novel mammalian reservoirs with increased human exposure risk. Concurrently, vaccination strategies are advancing beyond traditional adjuvanted inactivated vaccines toward next-generation platforms, including mRNA and virus-like particle vaccines, designed for rapid deployment and broader immune protection. However, ongoing viral evolution, constrained vaccine availability, and gaps in coordinated surveillance underscore the urgent need for an integrated One Health approach to reduce panzootic risk.

## 1. Introduction

Highly Pathogenic Avian Influenza (HPAI) virus subtype H5N1 stands as a prominent example of a continuously evolving zoonotic pathogen at the volatile interface of animal, human, and environmental health. Since its initial recognition as a direct threat to human health in 1997 [1], the epidemiological landscape of the virus has been rewritten. Dominated by clade 2.3.4.4b, the current era has traded sporadic cases for a persistent, global panzootic. This shift represents a ‘new normal’ where the virus maintains a constant presence in animal populations, stretching its ecological footprint further than ever recorded [2,3]. This shift represents a fundamental change in the virus’s behavior, moving beyond agricultural systems to cause mass mortality in wild birds and recurrent spillover into an expanding roster of mammalian hosts, including, most recently, dairy cattle [4].

The context and relevance of HPAI H5N1 research have become progressively urgent. The virus is a member of the *Orthomyxoviridae* family, possessing a segmented, negative-sense RNA genome that facilitates rapid evolution through mutation and reassortment [5]. Its pathogenicity and host range are primarily dictated by two surface glycoproteins: hemagglutinin (HA), responsible for host cell attachment and entry, and neuraminidase (NA), essential for viral egress [6]. The cardinal molecular feature distinguishing HPAI from low-pathogenic strains is the presence of a polybasic cleavage site (PCS) within the HA protein. This motif allows proteolytic activation by universal furin-like proteases present in most host tissues, enabling systemic, multi-organ infection rather than localized respiratory or enteric disease, and underpinning the extreme virulence observed in poultry [7].

Beyond its virological characteristics, HPAI H5N1 is a transboundary animal disease of the highest order, with cascading implications for global food security, economic stability, and public health [8]. The agricultural impact is staggering; control efforts have necessitated the culling of hundreds of millions of birds worldwide, causing severe supply chain disruptions and economic losses measured in billions of dollars. From a public health perspective, H5N1 remains one of the most concerning influenza viruses with pandemic potential [9]. While human infections remain sporadic and largely tied to direct animal contact, the case fatality rate (CFR) historically exceeds 50%, a stark contrast to seasonal influenza. The virus’s unchecked circulation in diverse animal reservoirs provides relentless opportunities for adaptation [10], increasing the risk of it acquiring the genetic changes necessary for efficient, sustained human-to-human transmission. Consequently, HPAI H5N1 serves as a critical model system for elucidating the principles of viral virulence, cross-species adaptation, and pandemic preparedness, demanding analysis through an integrated One Health framework that connects wildlife, agriculture, and human medicine [11].

The historical emergence and evolution of HPAI H5N1 trace a clear arc of increasing global threat. The progenitor of all contemporary viruses is the A/goose/Guangdong/1/1996 (Gs/Gd) lineage. While earlier H5 viruses were documented (e.g., in Scotland, 1959), the Gs/Gd lineage marked a novel, highly pathogenic evolutionary branch [12]. The virus catapulted onto the global health stage during the 1997 Hong Kong outbreak, which provided the first unequivocal evidence that a purely avian influenza virus could cause severe, fatal respiratory illness in humans without requiring passage through an intermediate host [1]. Aggressive poultry culling contained this initial event, but the virus re-emerged endemically in Asia in 2003, embarking on a wave of spread across continents via migratory bird flyways [13].

This period of geographic expansion was accompanied by intense genetic diversification. Through antigenic drift and shift, the original lineage splintered into multiple clades (0–9) and subclades, adapting to local avian populations and occasionally spilling over into humans, as seen persistently in Egypt [14]. A pivotal evolutionary leap occurred with the emergence of clade 2.3.4.4b in late 2020. This clade exhibited distinct traits including a propensity for efficient transmission among wild waterfowl and a characteristic long NA stalk that fueled an explosive and persistent panzootic. In late 2021 [15], these viruses traversed the Atlantic, likely via migratory birds, initiating North America’s most severe poultry outbreak. The spread continued unabated, reaching South America and, in a landmark event, Antarctica in late 2023, signifying its presence on all inhabited continents [16].

**Figure 1:**
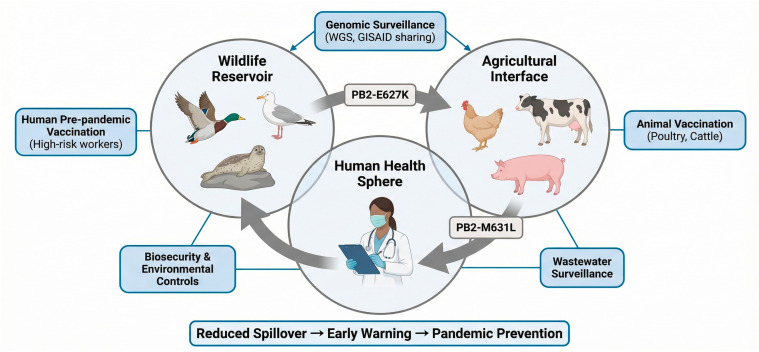
Integrated One Health Framework for HPAI H5N1 Surveillance and Mitigation. **Note**: Integrated One Health Framework for HPAI H5N1 Surveillance and Mitigation. Schematic representation of the interconnected spheres requiring coordinated action: Wildlife Reservoir (wild birds, marine mammals), Agricultural Interface (poultry, dairy cattle, swine), and Human Health. Gray arrows indicate viral spillover pathways, with key mammalian-adaptive mutations (PB2-E627K, PB2-M631L) noted at relevant interfaces. Blue intervention boxes represent core strategies: Genomic Surveillance (including whole-genome sequencing [WGS] and data sharing via GISAID), Animal Vaccination, Human Pre-pandemic Vaccination (for high-risk groups), and Biosecurity/Environmental Controls. Effective implementation of this framework aims to reduce viral amplification at source, detect adaptations early, and prevent pandemic emergence. Abbreviations: WGS, whole-genome sequencing; GISAID, Global Initiative on Sharing All Influenza Data.

The 2024 epizootic of HPAI H5N1 in U.S. dairy cattle (genotype B3.13) represents the most significant and recent development, signaling a dangerous new phase. (Note: This event post-dates many provided citations; expert analysis from USDA/CDC reports in 2024 would be cited here [17]). This outbreak, affecting over 200 herds, demonstrated efficient cow-to-cow transmission via milking equipment and revealed high viral loads in milk [17]. It clearly established cattle as a susceptible mammalian host, creating a novel and concerning interface for human exposure (e.g., among farm workers) and offering the virus a new environment in which to adapt to mammalian physiology. Concurrently, detections in over 70 wild and domestic mammalian species, from foxes to marine mammals, underscore a broadening host range that challenges existing surveillance and control paradigms [18,19]. The interconnected nature of this panzootic and the required integrated response are illustrated in Figure 1.

Leading experts now warn that HPAI H5N1 has transitioned from an agricultural crisis to an enduring ecological and zoonotic threat. The “emerging reality of the virus” is characterized by endemic circulation in wild bird reservoirs, constant pressure on poultry sectors, and recurrent, unpredictable spillover into mammals. This reality demands a paradigm shift in preparedness, moving from reactive culling towards proactive, science-driven strategies encompassing enhanced genomic surveillance, next-generation vaccine development for animals and high-risk humans, and robust One Health coordination. This review synthesizes the latest molecular insights into H5N1 pathogenicity and host adaptation, critically evaluates advances and challenges in vaccination, and frames these findings within the urgent context of contemporary panzootic risk mitigation [19].

## 2. Global Impact on Poultry, Livestock, and Public Health: An Escalating Multisectoral Crisis

HPAI H5N1 clade 2.3.4.4b virus has transcended its original ecological niche, triggering a persistent panzootic a pandemic in animals with profound and cascading consequences for global agriculture, wildlife ecosystems, and public health security. This transition from sporadic outbreaks to continuous, widespread circulation represents a fundamental shift in the virus’s epidemiology, demanding a reassessment of its long-term economic and zoonotic threat [20].

### 2.1. Catastrophic Impact on Poultry and Agricultural Systems

The poultry industry has borne the most immediate and severe economic brunt. In North America, the incursion that began in late 2021 is the largest and most severe on record. While initial reports cited substantial losses of domestic poultry in the United States by early 2023 (see Table 1 for detailed figures) [21,22], the cumulative impact in Canada, as of late 2025, has reached millions of birds affected across the country—underscoring the prolonged nature of the crisis [23].

In Europe, the 2021–2022 season saw over 40 million birds culled across 37 countries, leading to severe market disruptions. The scale of infection has caused significant volatility in global food commodity markets, with instability in the price of poultry and eggs, exposing critical vulnerabilities in centralized production systems and international supply chains [24,25].

The use of vaccination in poultry, as practiced in several countries including China, Egypt, and parts of Europe, presents a complex trade-off. While it can reduce clinical illness and mortality in flocks, it often does not confer sterilizing immunity, potentially allowing for “silent” circulation of the virus in vaccinated populations. This complicates surveillance, delays outbreak detection, and may create conditions for ongoing viral evolution, posing a significant challenge to long-term control strategies [26].

**Table 1:** Epidemiological, Molecular, and Public Health Features of the HPAI H5N1 Clade 2.3.4.4b Panzootic.

Category	Key Feature/Impact	Evidence/Example	Implication	Selected Refs.
Epidemiology	Transition to persistent global panzootic	Year-round circulation in wild birds across 6 continents	Fundamental epidemiological shift requiring long-term management	[20,21]
Agricultural Impact	Massive poultry losses	>58 M birds in US (2021–2023); ~17.2 M in Canada (2025)	Economic damage > $3B globally; supply chain vulnerability	[22,23,24,25]
Host Range Expansion	Infection of >70 mammalian species	Dairy cattle (B3.13 genotype), seals, foxes, minks	Creates new ecological niches and human exposure pathways	[27,28,29]
HA Polybasic Cleavage Site	Enables systemic infection	RERRRKR/G motif cleaved by ubiquitous proteases	Primary determinant of high pathogenicity in birds	[30,31]
HA Receptor Binding	Preference for avian (α2,3-SA) receptors	Q222L/G224S mutations can shift preference to human (α2,6-SA)	Major barrier to sustained human transmission	[32,33]
Polymerase Adaptation	Mammalian-adaptive mutations	PB2-E627K (enhances replication at 33 °C); PB2-M631L (cattle-associated)	Critical for host range expansion; detected in human cases	[34,35,36]
Zoonotic Transmission	High CFR but limited human spread	~50% CFR among confirmed cases; 2024 cattle outbreak added new exposure routes	Severe individual outcome but limited sustained transmission to date	[37,38]
Vaccination Strategy	Advancing next-generation platforms	mRNA vaccines (rapid redesign); universal HA stalk targets	Potential for more agile pandemic response but scale-up challenges remain	[39,40,41,42]

### 2.2. Significant Expansion into Livestock and Mammalian Wildlife

A defining and alarming feature of the current panzootic is the virus’s expanding host plasticity. H5N1 has now been confirmed in over 70 wild and domestic mammalian species, including red foxes, striped skunks, domestic cats, and marine mammals like seals and sea lions [43]. These repeated spillover events indicate the virus is successfully infecting a broad range of new hosts, with mass mortality events in South American marine mammals highlighting its devastating ecological impact [27].

The 2024 spillover into North American dairy cattle marked a fundamental shift in the virus’s behavior, moving it from a seasonal avian threat to a year-round livestock challenge. This epizootic affected over 200 herds across multiple states, where the outbreak (primarily genotype B3.13) revealed efficient cow-to-cow transmission linked to milking procedures. Critically, the virus demonstrated a distinct mammary gland tropism, replicating to high titers in milk [28,29]. This high concentration in raw milk suggests that the infection was being spread via contaminated milking equipment rather than traditional respiratory pathways. These findings established cattle as a significant new mammalian reservoir and a novel, high-volume pathway for potential human exposure [37,44].

**Figure 2:**
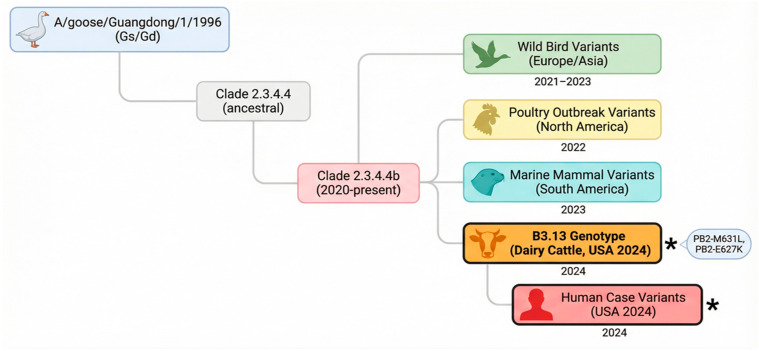
Simplified Phylogenetic Tree of HPAI H5N1 Clade 2.3.4.4b Evolution. **Note:** Simplified Phylogenetic Tree of HPAI H5N1 Clade 2.3.4.4b Evolution. Phylogenetic relationships of major HPAI H5N1 clade 2.3.4.4b variants circulating during the current panzootic. The tree highlights the emergence of the B3.13 genotype responsible for the 2024 dairy cattle outbreak in the United States and subsequent human infections. Branch lengths are schematic and not to scale. Asterisks (*) denote isolates in which specific mammalian-adaptive mutations have been detected: PB2-M631L (cattle-associated) in B3.13 genotype isolates, and PB2-E627K (broad mammalian adaptation) in human case isolates linked to cattle exposure. The tree illustrates the viral lineage diversification accompanying host range expansion and the acquisition of adaptive mutations at key nodes.

From a public health standpoint, the human infections associated with these dairy farms have been equally telling. Most patients identified during this period presented with mild conjunctivitis, a localized infection likely caused by direct mucosal contact with infected milk, rather than severe respiratory disease [38]. While these cases have remained mild so far, the presence of the PB2-E627K mutation in some isolates is a clear warning sign (Figure 2). It shows the virus is actively ‘retooling’ its machinery to replicate more efficiently in mammalian cells, making the dairy sector a critical frontline for both genomic surveillance and One Health coordination [19].

Further detections in species like goats and alpacas, along with the ever-present risk of infection in pigs—the classic ‘mixing vessel’ hosts due to their co-expression of avian and human-type influenza receptors—continuously raise the threat of viral reassortment. Such an event could combine the high virulence of H5N1 with the efficient transmissibility of human seasonal strains, generating a potential pandemic virus [29].

### 2.3. Evolving Public Health and Zoonotic Threat

Human infections remain sporadic but concerning. As of March 2025, the World Health Organization (WHO) has documented nearly 1000 confirmed human cases across two dozen countries since 2003, with an approximate case fatality rate (CFR) of 50% [44]. Most cases are linked to direct contact with infected birds or contaminated environments, though the 2024 cattle outbreak has added exposure to sick cattle or unpasteurized (raw) milk as a significant new risk factor [28,37].

Human clinical presentation appears increasingly diverse in the clade 2.3.4.4b era. Some recent cases in the U.S. and Europe, often linked to specific exposure routes (e.g., ocular splash), have presented as mild conjunctivitis [38], while severe and fatal pneumonia cases continue to be reported in other regions.

These observations may reflect localized reporting biases and active surveillance of exposed workers rather than a global shift in virulence. Meanwhile, severe pneumonia cases continue to be reported in regions like Cambodia and Chile, indicating that severity remains highly contingent on viral sub-lineage, inoculation route, and host factors. This variability necessitates more nuanced risk communication and flexible clinical preparedness.

A major biological constraint on a pandemic remains the virus’s receptor-binding preference. Most circulating H5N1 strains retain a strong affinity for α2,3-linked sialic acid receptors, prevalent in the human lower respiratory tract, rather than the α2,6-linked receptors dominant in the upper airway, which are necessary for efficient human-to-human transmission [45]. However, the virus’s widespread circulation in mammals increases opportunities for it to acquire the mutations needed to overcome this barrier. The key epidemiological and molecular features are summarized in Table 1.

## 3. Molecular Determinants of H5N1 Pathogenicity and Host Adaptation

The high virulence and expanding host range of HPAI H5N1 are orchestrated by a complex interplay of viral gene products that dictate host cell entry, replication efficiency, and evasion of immune defenses. Understanding these molecular mechanisms is critical for risk assessment, therapeutic design, and pandemic preparedness.

### 3.1. Hemagglutinin (HA): The Gatekeeper of Infection

The HA surface glycoprotein is the primary determinant of tissue tropism and systemic spread.

- Polybasic Cleavage Site (PCS): The hallmark of HPAI viruses is a polybasic cleavage site (e.g., RERRRKR/G) in the HA protein. Unlike LPAI strains, which require trypsin-like proteases found only in specific tissues, the H5 PCS is cleaved by ubiquitous furin-like proteases present throughout the host body. This enables systemic viral replication, leading to disseminated infection and multi-organ failure in birds [30,32].
- Receptor Binding Domain Structure: The HA receptor binding site is configured to preferentially accommodate sialic acid (SA) receptors with α2,3 linkages. Key amino acid positions (residues 222 and 224 in H5 numbering) form the binding pocket that determines this specificity. Mutations at these positions (Q222L, G224S) can alter the binding pocket conformation to favor α2,6-linked receptors, though such changes may compromise HA stability [31].
- HA Stability and Membrane Fusion: The pH threshold at which HA undergoes conformational change to trigger membrane fusion is determined by specific residues. Mutations that lower this pH threshold (e.g., T318I) enhance the acid stability of the HA protein, which is necessary for survival in acidic environments such as the human upper respiratory tract [33].

### 3.2. Polymerase Complex: The Engine of Replication and Adaptation

The viral RNA-dependent RNA polymerase (composed of subunits PB2, PB1, and PA) is a major driver of host range restriction and adaptation.

- Key Mammalian-Adaptive Mutations:
  ○ PB2-E627K: This is the most recognized marker, dramatically enhancing polymerase activity at the cooler temperatures (~33 °C) of the mammalian upper respiratory tract [46].
  ○ PB2-D701N: This mutation improves the binding of the viral ribonucleoprotein complex to mammalian importin-α isoforms, facilitating nuclear import and replication in human cells [34].
  ○ PB2-M631L: Notably identified in >99% of sequences from the 2024 U.S. dairy cattle outbreak, this mutation is strongly associated with viral adaptation in this novel mammalian host and has been found in subsequent human cases linked to cattle exposure [35].
- Host Cofactor Interaction: New research into host cofactors has shed light on why it is so difficult for this virus to jump between species. Essentially, the bird flu virus needs a specific host protein called ANP32A to copy itself. However, the bird version of this protein has an extra 33-amino acid ‘tail’ that the human version lacks. This structural difference acts like a lock that an unadapted virus cannot open. For the virus to replicate in humans, it usually needs a specific mutation PB2-E627K which reshapes the viral machinery to work with the shorter human version of the protein [36].

Furthermore, human cells express intrinsic restriction factors such as BTN3A3, which can inhibit replication of avian-origin influenza viruses. Surveillance studies have identified mutations in circulating H5N1 strains (e.g., N52D in the nucleoprotein) that may confer evasion of BTN3A3 restriction, representing another layer of adaptive evolution requiring close monitoring [36,47].

### 3.3. Non-Structural Proteins: Masters of Immune Evasion

Proteins NS1 and PB1-F2 are crucial for subverting the host’s first line of defense, the innate immune system.

- NS1—Interferon Antagonism: The NS1 protein is a potent multifunctional interferon (IFN) antagonist. It inhibits the production and signaling of type I IFNs, crippling the host’s antiviral state. Specific mutations, such as D92E, enhance this function by conferring resistance to the antiviral effects of both IFNs and TNF-α [48,49].
- PB1-F2—Pro-apoptotic Activity: The PB1-F2 protein, particularly with the N66S mutation, contributes to virulence by inducing apoptosis in immune cells like macrophages and lymphocytes, thereby depleting the host’s immune response capacity and exacerbating inflammation [50].

### 3.4. Neuraminidase (NA) and Accessory Factors

- NA’s Role in Viral Fitness: NA facilitates viral egress by cleaving sialic acids from the host cell and nascent virions. An optimal functional balance between HA binding and NA cleavage is required for efficient replication. The long NA stalk characteristic of clade 2.3.4.4b viruses is associated with increased fitness in wild birds and may facilitate infection in mammals [51,52].
- Accessory Protein Contributions: Other viral components support adaptation. Mutations in the Nucleoprotein (NP), such as N319K, synergize with PB2 mutations to enhance vRNP import, while others (e.g., N52D) help the virus evade human-specific restriction factors like BTN3A3 [47,53].

### 3.5. Host-Driven Pathogenesis: The Cytokine Storm

A critical determinant of severe disease in mammals is the dysregulated host immune response. H5N1 infection of respiratory epithelial and endothelial cells can trigger a hyper-cytokinemia (“cytokine storm”), characterized by an excessive release of pro-inflammatory cytokines like IL-6, TNF-α, and IFN-γ [54,55]. This excessive inflammation, more so than direct viral cytopathic effect, is a primary driver of the severe alveolar damage, acute respiratory distress syndrome (ARDS), and multi-organ failure observed in fatal human cases. This pathogenesis underscores why broad-spectrum immunomodulatory therapies are being investigated as adjuncts to antiviral treatment [56].

## 4. Zoonotic Potential and Cross-Species Adaptation: Navigating a Shifting Risk Landscape

The zoonotic potential of HPAI H5N1 is not static but is actively shaped by the virus’s evolution within an expanding host range. The significant spread of clade 2.3.4.4b has transformed this potential from a theoretical concern into a tangible (Figure 2), ongoing global challenge [57]. Experts now emphasize that the traditional model of sporadic bird-to-human spillover has been superseded by a more complex reality involving persistent environmental reservoirs, novel mammalian hosts, and new exposure pathways, all of which increase the frequency of human-virus encounters and opportunities for adaptation [58].

### 4.1. Receptor Binding Specificity and Tissue Tropism: The Fundamental Barrier

Building on the molecular features of HA discussed in Section 3.1, the receptor binding specificity of the viral hemagglutinin represents the primary determinant of host range restriction. The preferential binding of avian-adapted viruses to α2,3-linked sialic acid receptors—abundant in the avian intestinal tract and, importantly, in the human lower respiratory tract—limits efficient infection of the human upper airway, where α2,6-linked receptors predominate [59,60,61]. This receptor distribution has direct clinical implications: binding to α2,3-SA receptors deep in the alveoli is associated with severe viral pneumonia, while ocular infection (conjunctivitis) is facilitated by the presence of α2,3-SA receptors in the human eye [62].

Mutations that shift binding preference toward α2,6-SA (such as Q222L and G224S, described structurally in Section 3.1) have been generated in laboratory settings and shown to confer airborne transmission in ferret models [33,63]. However, their natural occurrence in circulating H5N1 strains remains rare, indicating that while the virus has the genetic potential to overcome this barrier, it has not yet done so in the field.

### 4.2. Pathways for Cross-Species Spillover: Ecological and Behavioral Drivers

Spillover is a multi-factorial event occurring at the intersection of viral opportunity, host ecology, and human activity.

- Novel Exposure Routes: While direct contact with infected poultry remains the dominant risk, the 2024 epizootic in U.S. dairy cattle created a paradigm shift. Exposure now includes contact with infected cattle, aerosolized milk during milking, and consumption of unpasteurized (raw) milk containing high viral loads, posing a risk to farm workers and consumers [29].
- The “Mixing Vessel” Hypothesis in Action: Species that express both avian (α2,3) and human (α2,6) SA receptors in their respiratory tracts are considered potential “mixing vessels.” While pigs are the classic example [64], minks in fur farms have proven to be exceptionally efficient hosts for H5N1, facilitating mammalian adaptation and intra-species transmission [27]. Dairy cattle, with receptor distribution still under investigation, now represent a critical new mammalian host where viral reassortment with endemic cattle or human seasonal viruses could theoretically occur [65].
- Environmental Amplification: The virus’s endemicity in wild bird populations leads to widespread environmental contamination of waterways, pastures, and feed. This creates persistent exposure risks for free-range poultry, livestock, and wild mammals, acting as a constant source for reintroduction to farms despite biosecurity measures [66].

### 4.3. Molecular Signatures of Mammalian Adaptation: Lessons from Recent Outbreaks

Genomic surveillance of infections in novel mammalian hosts provides a real-time window into adaptive evolution:

- Dairy Cattle (2024): A defining mutation, PB2-M631L, was found in >99% of sequences from infected U.S. cattle, strongly associating it with replication in this host. Even more concerning, the well-known mammalian-adaptive marker PB2-E627K was identified in a human case linked to cattle exposure, demonstrating that viruses pre-adapted to mammals are already reaching humans [36].

The 2024 multi-state epizootic in U.S. dairy cattle represents a paradigm shift in H5N1 ecology. Characterized by the B3.13 genotype, this outbreak demonstrated efficient cow-to-cow transmission linked to milking procedures and revealed a distinct mammary gland tropism with high viral loads in milk (>10^6^ TCID50/mL) [29]. The near-ubiquitous presence of the PB2-M631L mutation (>99% of sequences) strongly associates with adaptation to this novel bovine host. Of grave concern, viruses from this lineage have infected humans: a dairy worker in Texas developed conjunctivitis after exposure to infected cattle, and the isolate contained both PB2-M631L and the classic mammalian-adaptive marker PB2-E627K [46]. This demonstrates that H5N1 viruses pre-adapted to mammals are already reaching human populations through new agricultural interfaces, with raw milk consumption posing an additional public health risk [29,38].

- Farmed Minks (2022–2023): Outbreaks on Spanish and Finnish fur farms revealed viruses acquiring mutations like PB2-T271A, which enhances polymerase activity in mammals and was also a feature of the 2009 H1N1 pandemic virus [67].
- Wild Carnivores and Marine Mammals: Frequent detection of PB2-E627K and D701N in foxes, seals, and sea lions confirms strong selective pressure for polymerase adaptations that enhance replication at mammalian body temperatures and improve nuclear import of the viral genome in mammalian cells [68].

### 4.4. Genetic and Biological Hurdles to a Pandemic

Despite these adaptations, H5N1 faces a high genetic barrier to achieving efficient, sustained human-to-human transmission. This barrier is polygenic:

- HA Stability Trade-off: Mutations that improve human-receptor binding (e.g., Q226L) often destabilize the HA protein, compromising its ability to survive the acidic environment of the human upper respiratory tract or triggering premature fusion. The virus must balance receptor avidity with structural stability [46].
- The Requirement for Coordinated Changes: Laboratory studies demonstrate that airborne transmission in ferrets requires a specific combination of mutations affecting HA receptor binding, HA stability, and polymerase efficiency (like PB2-E627K). The concurrent emergence of this exact set in nature remains a low-probability event, though the expanding viral population in mammals increases the odds [69].
- Host Intrinsic Immunity: Human cells possess innate restriction factors like MxA and BTN3A3 that potently inhibit avian-origin influenza polymerases. H5N1 must acquire further compensatory mutations (e.g., in the Nucleoprotein NP) to evade these defenses, adding another layer to the adaptive challenge [70].

### 4.5. A One Health Imperative: Integrated Surveillance and Risk Mitigation

Managing this evolving threat demands a unified One Health approach integrating human, animal, and environmental health surveillance (Figure 1).

- Targeting High-Risk Interfaces: Live Bird Markets (LBMs) have been repeatedly identified as epicenters for viral amplification, reassortment, and human infection. Interventions like market closures, mandatory rest days, and banning live poultry overnight were highly effective in eliminating human H7N9 cases in China and remain a critical control measure [71].
- Deploying Innovative Surveillance: Deploying Innovative Surveillance: Wastewater-based surveillance (WWS), while highly effective during the COVID-19 pandemic, remains an emerging yet high-potential tool for H5N1. While it offers a non-invasive way to monitor communities and dairy sites, further research is needed to fully standardize its role as a definitive early warning signal [72].
- Addressing Critical Gaps: Significant surveillance blind spots remain due to geographic inequities in sequencing capacity, under-reporting in backyard farms and wildlife, and a lack of systematic monitoring of potential intermediary hosts. Strengthening the Global Influenza Surveillance and Response System (GISRS) framework to better incorporate animal and environmental data is a stated priority for closing these gaps [73].

## 5. Advances in Vaccination Strategies: Bridging Preparedness and Reality

Vaccination remains the cornerstone of pandemic preparedness, but the rapid evolution and ecological spread of H5N1 demand a re-evaluation of traditional strategies. The goal has shifted from simply stockpiling a matched vaccine to developing agile platforms capable of rapid deployment and eliciting broad, durable immunity against a moving target.

### 5.1. Traditional and Stockpiled Human Vaccines

Current preparedness relies on inactivated, adjuvanted vaccines, which are proven safe and form the basis of national stockpiles.

- Licensed Platforms: Vaccines like AUDENZ^®^ (approved by the U.S. FDA in 2020 for persons 6 months and older) and Adjupanrix^®^ (EU-approved) are matched to older H5N1 clades but can provide a priming immune response [19]. Their production uses established egg- or cell-based technologies, but a switch to a new strain still requires 4–6 months for significant dose production [74].
- The Scale Challenge: A critical vulnerability is insufficient global manufacturing capacity. For example, the U.S. stockpile of pre-pandemic H5 vaccine would only cover a fraction of the population, highlighting a major gap between preparedness plans and production reality [75].

### 5.2. Next-Generation Vaccine Platforms

The evolving landscape of H5N1 vaccine development encompasses multiple technological platforms, each with distinct advantages and limitations (Table 2). Traditional egg-based vaccines remain the backbone of pandemic stockpiles but face constraints in speed and scalability. Next-generation platforms, particularly mRNA-LNP (mRNA-lipid nanoparticle) vaccines, offer unprecedented rapid response capabilities potentially reducing vaccine development timelines from months to weeks though challenges in thermostability and delivery infrastructure persist [76,77]. The optimal preparedness strategy likely involves a ‘portfolio approach’ maintaining diverse platform capabilities.

Innovation focuses on speed, breadth of protection, and dose-sparing.

- mRNA-LNP Vaccines: Building on COVID-19 successes, nucleoside-modified mRNA platforms offer the fastest potential response. The U.S. HHS has invested heavily (e.g., $176 million to Moderna in 2024) to advance these vaccines, which can be redesigned and produced at scale within weeks [76]. Early candidates against 2.3.4.4b show promise in animal models, but their efficacy in humans against avian influenza remains unproven [77].
- Virus-Like Particles (VLPs) and Recombinant Proteins: VLPs, which mimic the virus structure without its genetic material, are highly immunogenic. Produced in insect cells or plants, they offer scalable and safe manufacturing alternatives [78].
- Broadly Protective Strategies: Research is actively pursuing universal vaccine targets, such as the conserved stalk region of HA or the M2e peptide, to provide protection across diverse H5N1 clades and even other influenza subtypes, potentially overcoming the problem of antigenic drift [79].

### 5.3. Animal Vaccination: Controlling the Source

Vaccination of animal reservoirs is a critical One Health strategy to reduce viral load at the source and minimize human exposure.

- Poultry: China’s mandatory bivalent H5/H7 poultry vaccination program successfully suppressed the H7N9 virus and eliminated human cases, demonstrating proof of concept [80]. However, sub-optimal vaccination that reduces disease without preventing infection can lead to “silent circulation,” driving antigenic drift and complicating surveillance a major concern for ongoing programs [81].
- Livestock: The 2024 cattle outbreak spurred urgent research. The USDA is evaluating H5N1 vaccine candidates for dairy cattle, aiming to reduce mammary gland infection, milk shedding, and economic loss. Key challenges include demonstrating efficacy, securing a viable vaccine market, and establishing a feasible delivery system for a previously unvaccinated livestock sector [39].
- High-Risk Human Groups: Some nations have begun proactive vaccination of at-risk individuals. Finland’s 2024 voluntary program for poultry and fur farm workers, using a vaccine matched to 2.3.4.4b, reported high immunogenicity but low uptake, underscoring the profound challenge of vaccine acceptance even among those with the highest exposure risk [40].

### 5.4. Comparative Analysis of Vaccine Platforms and Challenges

Several H5N1 vaccine candidates are progressing through clinical evaluation, reflecting intensified pandemic preparedness efforts following the 2024 panzootic expansion (Table 3). Notably, mRNA-1010 (Moderna) represents the first mRNA-based H5N1 candidate to enter Phase 1/2 trials, with interim results expected in 2025 [76]. Concurrently, established platforms continue to advance, with Seqirus’s adjuvanted inactivated vaccine completing Phase 3 immunobridging studies to support regulatory authorization for new clades [19]. This diversified pipeline underscores the global commitment to reducing the timeline from virus identification to vaccine availability.

## 6. Molecular Surveillance and Predictive Modeling: The Digital Front Line

The modern response to H5N1 is increasingly data-driven, relying on advanced genomics and computational modeling to anticipate threats and guide interventions [41].

### 6.1. Genomic Surveillance and Open Data

- Whole-Genome Sequencing (WGS): WGS is indispensable for tracking transmission pathways, identifying reassortment events, and detecting adaptive mutations like PB2-E627K or HA receptor-binding changes in near real time [42].
- Bioinformatics Pipelines: Tools like INSaFLU automate the workflow from sequence data to actionable reports, enabling public health labs to perform detailed genomic surveillance [82].
- Global Data Sharing: Platforms like GISAID provide the essential infrastructure for the rapid, open sharing of virus sequences with attribution. This collaborative model, which accelerated during COVID-19, is vital for tracking the global movement and evolution of H5N1 and for selecting appropriate Candidate Vaccine Viruses (CVVs) [83].

Deploying Innovative Surveillance: We can significantly sharpen our early warning systems by utilizing wastewater-based surveillance (WWS). This approach was a game-changer during the COVID-19 pandemic, and it is now proving to be a high-potential frontier for H5N1 monitoring [19]. Because it is non-invasive and cost-effective, WWS allows us to ‘listen’ for viral signals in human communities and near high-risk sites like dairy processing plants before outbreaks even become visible. While we have already seen that detecting H5N1 fragments in environmental runoff is entirely feasible, the method is currently in a transition phase moving from promising research to a validated, standardized tool within our national biosecurity frameworks [19].

### 6.2. Risk Assessment and Predictive Analytics

- Standardized Risk Frameworks: Tools like the WHO’s Tool for Influenza panzootic Risk Assessment (TIPRA) use standardized criteria (e.g., virus properties, population immunity, ecology) to assign risk scores to emerging viruses, helping prioritize resources [84].
- In silico Phenotype Prediction: Computational methods, including machine learning and algorithms like the Informational Spectrum Method (ISM), can analyze sequence data to predict functional traits such as increased human receptor binding, offering early warnings from genomic data alone [85].
- Transmission and Intervention Modeling: Mathematical models simulate outbreak dynamics to answer critical policy questions. For example, models can project the course of an outbreak in dairy cattle or compare the potential efficacy of vaccination versus culling in poultry, informing cost-effective control strategies [86].

## 7. Critical Knowledge Gaps and Future Research Priorities

Despite advances, persistent uncertainties hinder optimal preparedness and require targeted research.

- Pathogenesis in Novel Hosts: The pathogenic mechanisms in cattle and the drivers of the severe neurotropism observed in cats and marine mammals are poorly understood and require urgent study [29].
- Quantifying Reassortment Risk: The probability of a pandemic strain emerging via reassortment in a “mixing vessel” like mink or cattle is considered high but is difficult to quantify. Enhanced surveillance at animal–human interfaces is needed to detect such events early [87].
- Improving Preclinical Models: The predictive value of ferret models for human vaccine efficacy and transmission remains imperfect. Developing more accurate models, including organoids and advanced in vitro systems, is crucial for reliable evaluation of countermeasures [88].
- Equity in Global Surveillance: Surveillance and sequencing capacity are overwhelmingly concentrated in high-income nations, creating dangerous blind spots in low- and middle-income regions where the virus may be evolving undetected. Building equitable, global genomic surveillance networks is a fundamental priority for pandemic prevention [89,90].

### Critical Synthesis: Bridging Molecular Insights to Public Health Action

The molecular determinants of H5N1 pathogenicity and adaptation discussed herein must translate into actionable public health strategy. The detection of specific mutations serves as more than academic observation it provides an early warning system. For instance, surveillance programs should prioritize sequencing for PB2-M631L in livestock and PB2-E627K in mammals as sentinel markers of increased zoonotic risk. Similarly, the identification of HA mutations affecting receptor binding (Q222L/G224S) or stability (T318I) should trigger enhanced vigilance at animal–human interfaces [32,33].

## 8. Strategic Imperatives: Navigating the New Era of H5N1

Recent studies have also explored mRNA-based vaccination approaches targeting H5 hemagglutinin for protection in mammalian hosts, including experimental evaluation in lactating dairy cattle, highlighting the expanding role of next-generation vaccine platforms in controlling emerging H5N1 infections across species [91].

### 8.1. Policy and Strategic Recommendations for a Proactive Defense

To mitigate this elevated and persistent threat, a reactive posture is inadequate. The global community must adopt a proactive, pre-emptive strategy built on the following interconnected pillars:

- Implement Genomic Surveillance as a Core Public and Animal Health Utility.

Routine, real-time Whole-Genome Sequencing (WGS) of viruses from human cases, livestock outbreaks, and wildlife mortality events must become the standard, not the exception. This is non-negotiable for tracking the emergence of high-consequence mutations (e.g., in HA receptor binding or polymerase complex) and detecting reassortment events [87]. This system must be underpinned by equitable global data sharing through platforms like GISAID, with immediate and transparent reporting to overcome the dangerous surveillance gaps that currently exist in many regions [83].

b. Operationalize the One Health Framework through Integrated Sentinel Systems.

Collaboration between human, animal, and environmental health sectors must move from rhetoric to integrated operational programs. This includes:

- Scaling Environmental Surveillance: Expanding wastewater surveillance to monitor sentinel locations (e.g., near large poultry facilities, dairy farms, and wildlife congregations) provides a cost-effective, population-level early warning system for viral incursion [72].
- Formalizing Wildlife and Livestock Sentinel Networks: Establishing targeted, risk-based virological and serological surveillance in high-risk species (e.g., pigs, peri-domestic carnivores like foxes, and marine mammals in affected areas) to act as an early detector of mammalian adaptation and spillback events [36].

c. Accelerate the Vaccine Ecosystem for Both Prevention and Response.

The vaccination strategy must be dual-track: preparing for a human pandemic while aggressively reducing the animal source.

- For Human Pandemic Preparedness: Investment must accelerate beyond traditional egg-based platforms. mRNA and recombinant nanoparticle vaccine technologies offer the speed and flexibility required to respond to a rapidly evolving threat or a sudden pandemic spark. Concurrently, sustained research into universal influenza vaccine candidates targeting conserved viral regions remains a critical long-term goal for overcoming antigenic drift [77,79].
- For Animal Reservoir Control: The development and strategic deployment of vaccines in animal reservoirs is a legitimate and necessary public health intervention. Learning from China’s success with H5/H7 poultry vaccination, the international community must support the rapid development and evaluation of effective vaccines for livestock, beginning with dairy cattle [39,80]. This must be coupled with robust “DIVA” (Differentiating Infected from Vaccinated Animals) strategies and surveillance to prevent masking virus circulation.

d. Enforce and Fundamentally Re-think Agricultural Biosecurity.

The scale of environmental viral contamination requires moving beyond farm-gate hygiene to systemic, sector-wide reforms. This includes critical assessments of high-risk practices, such as the density of large-scale production near key migratory bird flyways or wetlands, and the structure of outdoor and free-range systems during high-risk periods. Innovation in engineering controls (e.g., air filtration for indoor poultry and livestock barns) and protection for farm workers through PPE and access to pre-pandemic vaccination must be prioritized.

e. Prioritize High-Risk Group Protection and Justified Use of Pre-pandemic Vaccines.

Given the clear occupational risk to poultry and dairy workers, veterinarians, and outbreak responders, nations should establish pre-positioned agreements and clear regulatory pathways for the rapid deployment of matched pre-pandemic vaccines to these groups during active outbreaks, as piloted in Finland. This is a direct barrier to zoonotic transmission and protects essential workers [40].

### 8.2. A Call for Sustained Vigilance and Global Solidarity

The history of H5N1 over the past three decades is a testament to the virus’s relentless capacity for evolutionary surprise. The recent expansion into dairy cattle is not a culmination but a warning of further unexpected adaptations to come. The goal of pandemic preparedness is not prediction but resilience, building the integrated surveillance, scientific, and public health infrastructure capable of detecting, understanding, and rapidly countering each new manifestation of the threat.

Achieving this requires a commitment to global solidarity and equitable resource sharing. The nations facing the greatest outbreak burdens are often those with the fewest resources for control. Strengthening capacity worldwide is not merely an ethical imperative but a strategic necessity to protect all. By integrating cutting-edge science, proactive policies, and genuine collaboration across the One Health spectrum, the global community can transition from a cycle of response to a state of prepared resilience, ready to meet the enduring challenge of HPAI H5N1 [72].

## 9. Conclusions

The Highly Pathogenic Avian Influenza (HPAI) H5N1 virus, particularly the clade 2.3.4.4b lineage, has undergone an ecological and epidemiological paradigm shift. It has evolved from a severe poultry disease managed through localized culling to a persistent global panzootic, now endemically circulating in wild bird populations across six continents and spilling over with alarming frequency into a vast and growing range of mammals [20,21]. The 2024 multi-state epizootic in U.S. dairy cattle a species previously considered an unlikely host serves as the most definitive signal that the traditional boundaries separating avian [90], wildlife, livestock, and human health spheres have fundamentally eroded [23]. As noted by global health agencies, this marks a transition to a more complex phase where managing endemic animal disease is directly synonymous with pandemic prevention [29].

While sustained human-to-human transmission has not been observed, the risk profile has demonstrably increased. The virus is now replicating in tens of millions of novel mammalian host cells daily, providing some significant incentive to acquire adaptive mutations. The detection of classic mammalian-adaptive markers like PB2-E627K in human cases linked to cattle exposure confirms that pre-adapted viruses are already reaching people through new interfaces [36]. The primary panzootic risk is no longer a single, sudden genetic leap in birds, but the cumulative probability of the virus piecing together the necessary adaptive traits from within a broad mammalian reservoir, a process experts describe as “evolution in real time” at an expanded animal–human interface [41].

## Figures and Tables

**Table 2:** Comparison of H5N1 Vaccine Platforms: Advantages, Limitations, and Current Status.

Platform	Typical Development Timeline	Key Advantages	Major Limitations	Current Development Status (2024–2025)	Representative Examples & References
Egg-based Inactivated	4–6 months	Proven safety record Established global manufacturing capacity Regulatory familiarity (well-established licensing pathways with decades of regulatory precedent)	Dependent on egg supply and suitability Slower strain update capability Potential for egg-adaptive mutations	**Licensed and** **stockpiled**	AUDENZ^®^ (Seqirus), licensed in US/EU for pre-pandemic use [19,75]
Cell-based Inactivated	3–4 months	No egg adaptation required More scalable than egg-based Faster initiation of production	Higher production costs Limited global manufacturing footprint Often requires adjuvant	**Licensed and** **stockpiled**	Celvapan (Baxter), pandemic preparedness stockpiles [74]
mRNA-LNP	**Weeks to 2 months**	Ultra-rapid design and manufacturing Flexible platform for multivalent formulations Strong Th1-biased immune response	Cold chain requirements (−20 °C to −80 °C) Reactogenicity profile concerns Limited long-term safety data for influenza	**Phase 1/2 clinical** **trials**	Moderna mRNA-1010 (NCT06197191); Pfizer/BioNTech candidates [76,77]
Virus-like Particle (VLP)	2–3 months	No viral genetic material Presents native conformational antigens Can incorporate multiple HA subtypes	Complex manufacturing process Scale-up challenges Higher cost of goods	**Preclinical to** **Phase 1**	Medicago plant-based VLP platform; Novavax Matrix-M adjuvanted [78]
Recombinant HA Protein	3–4 months	High purity and consistency No infectious virus handling Stable formulation	Often requires potent adjuvants May require multiple doses Lower immunogenicity than whole virus	**Clinical** **development**	Sanofi recombinant HA vaccines; GSK pandemic candidates [79]
Viral Vector (e.g., Adenovirus)	2–3 months	Strong cellular immune induction Single-dose potential Thermostable formulations possible	Preexisting immunity may reduce efficacy Safety concerns in certain populations Limited H5N1-specific development	**Early clinical/** **preclinical**	Janssen Ad26 platform; University of Oxford ChAdOx1 [79]

**Notes:** 1. “Regulatory familiarity” refers to the extensive experience that regulatory agencies (FDA, EMA, etc.) have accumulated over decades with egg-based inactivated influenza vaccines, resulting in well-established licensing pathways and predictable review timelines. 2. “No egg adaptation required” indicates that cell culture-based methods use mammalian cell lines, avoiding the egg-adaptive mutations that can alter viral antigenicity and potentially reduce vaccine match to circulating strains. 3. All inactivated vaccines (egg-based and cell-based) typically require adjuvants for optimal immunogenicity, particularly for pandemic preparedness where dose-sparing is critical. 4. “Reactogenicity concerns” for mRNA vaccines refer to higher rates of injection site reactions, fever, and fatigue observed in clinical trials compared to traditional inactivated vaccines; these effects are typically dose-dependent and self-limiting. 5. **Abbreviations:** LNP, lipid nanoparticle; HA, hemagglutinin; VLP, virus-like particle; FDA, Food and Drug Administration; EMA, European Medicines Agency.

**Table 3:** Select H5N1 Vaccine Candidates in Clinical Development (2024–2025).

Name	Platform Technology	Target Antigen/Clade	Developer/ Sponsor	Current Clinical Stage	Clinical Trial Identifier (NCT)/Key Reference
mRNA-1010	Nucleoside-modified mRNA-LNP	H5 HA (clade 2.3.4.4b)	Moderna, Inc.	Phase 1/2 (interim)	NCT06197191; Moderna press release (2024) [76]
VRBP-200	Recombinant HA (insect cell)	H5 HA (clade 2.3.4.4b)	Sanofi Pasteur	Phase 1/2 (completing)	Company pipeline; expected 2025 data
GLS-5310	DNA plasmid + electroporation	H5 HA + conserved NP/M2e	GeneOne Life Science, Inovio	Phase 1 (completed)	NCT05639335; reported immunogenicity (2024)
Cohort 1: Adjuvanted Inactivated	Egg-based, MF59-adjuvanted	H5 (clade 2.3.4.4b)	Seqirus (CSL)	Phase 3 immunobridging	Part of US prepandemic stockpile; licensed 2020 [19]
MVC-COV1901 (H5 variant)	Protein subunit (S-2P)	H5 HA (multiple clades)	Medigen Vaccine Biologics	Phase 1 (initiated)	Based on successful COVID-19 platform; 2024 announcement
VXA-H5-1.1	Adenovector (oral)	H5 HA + dsRNA adjuvant	Vaxart	Phase 1 (planned)	Oral tablet format; preclinical data 2023
INfluenza-VLPs	Plant-derived VLP	H5 HA (2.3.4.4b)	Medicago (GSK)	Preclinical Phase 1	Platform validated for seasonal flu; H5N1 in development [78]

## Data Availability

No new data were created or analyzed in this study. Data sharing is not applicable to this article.
